# Hookworm infection in central China: morphological and molecular diagnosis

**DOI:** 10.1186/s13071-021-05035-3

**Published:** 2021-10-14

**Authors:** Fang Fang Xu, Yu Fei Niu, Wen Qing Chen, Sha Sha Liu, Jing Ru Li, Peng Jiang, Zhong Quan Wang, Jing Cui, Xi Zhang

**Affiliations:** Department of Parasitology, School of Basic Medical Sciences, Zhengzhou University, Zhengzhou, 450001 China

**Keywords:** Hookworm, *Necator americanus*, Ancylostomiasis, Diagnosis, Mitochondrial genome

## Abstract

**Background:**

*Necator americanus* is one of the major etiological agents of human ancylostomiasis. Historically, the epidemiology of ancylostomiasis in Henan Province of central China and the molecular characteristics of *N. americanus* have been poorly understood.

**Methods:**

In this study, we report a case of ancylostomiasis in Zhengzhou city of Henan Province. We also review the epidemiology of ancylostomiasis in Henan Province from 1949 to 2020. In addition, the complete mitochondrial (mt) genome of one clinical isolate is fully characterized using Illumina sequencing. All available mt genomes of hookworms in GenBank were included to reconstruct the phylogeny using both maximum likelihood (ML) and Bayesian inference (BI) methods.

**Results:**

A total of three worms were collected from the patient. These worms were identified as *N. americanus* based on morphological characteristics as well as confirmed by genotyping with the barcoding gene *cox*1. Although ancylostomiasis cases have dropped substantially in recent years, hookworm infection is still a public health problem in underdeveloped areas and remote rural areas in Henan Province. The mt genome features of the *N. americanus* contained 12 protein-coding genes (PCGs), 22 transfer RNA genes, two ribosomal RNA genes, and a major non-coding region. The *nad*1 gene showed high sequence variability among isolates, which is worth considering for future genetic studies of *N. americanus*. Phylogenetic analyses support the monophyly of hookworm isolates from different hosts and distinct geographical locations.

**Conclusions:**

The mt genome of *N. americanus* presented here will serve as a useful data set for studying population genetics and phylogenetic relationships of hookworms. Positive measures for preventing and controlling ancylostomiasis are required by both health services and individuals in Henan Province.

**Graphical abstract:**

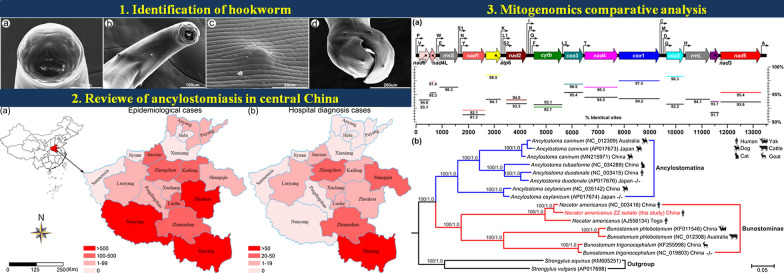

**Supplementary Information:**

The online version contains supplementary material available at 10.1186/s13071-021-05035-3.

## Background

Human hookworms are geohelminths, causing physical and intellectual growth retardation and sometimes fatal iron deficiency anemia [1]. Hookworm infections are regarded as one of the most important neglected tropical diseases, with over 500 million people infected worldwide [2]. The majority of human ancylostomiasis cases are caused by the species *Ancylostoma duodenale* or *Necator americanus* [3]. *Necator americanus* is prevalent in the tropics and subtropics, while *A. duodenale* tends to live in dry and cold climates [4]. In China, ancylostomiasis not only significantly impairs the health of people but also leads to great economic losses [5]. The vast rural areas of Henan Province in central China are the main endemic regions of ancylostomiasis. However, little is known about the prevalence, infection dynamics, and genetic diversity of hookworms in the area.

In clinical diagnostics, it is difficult to perform accurate species identification of hookworms when the collected worm is incomplete, especially when key taxonomic characters in the head or tail are damaged. In contrast, the DNA barcoding approach has been useful for hookworm identification [6]. Several DNA markers have been applied in the taxonomic and genetic studies of hookworms, including the cytochrome *c* oxidase subunit 1 gene, *cox*1 [7], and the first internal transcribed spacer of ribosomal DNA, ITS1 [8]. However, in comparison with a single gene, the complete mitochondrial (mt) genome can provide more markers for molecular identification, and the potential to discover population variants or cryptic species [9]. Several complete mitochondrial DNA (mtDNA) sequences of hookworms have been sequenced and characterized, such as *N. americanus*, *A. duodenale*, *A. caninum*, *A. ceylanicum*, and *A. tubaeforme* [10–12]. These available mtDNA data sets offer the opportunity to compare the diversity of mt genomes among closely related taxa.

This study reports a case of ancylostomiasis in central China and identifies the isolated hookworms using morphological and molecular methods. One of the clinical isolates was sequenced using next-generation sequencing (NGS) technology to perform a mitogenomics comparative analysis of hookworms and to discuss the implications of the data set as a new resource for future genetic studies.

## Methods

### Morphological and molecular identification

The patient’s fecal samples were examined by the Kato-Katz thick smear technique using light microscopy (Olympus CX23, Olympus Corporation, Japan). Under gastroduodenoscopy, hookworms were collected in the duodenal bulb of the patient using endoscopic biopsy forceps. The collected worms were then washed in water and stored in 70–80% ethanol until morphological and molecular analyses. The morphological study of hookworms was performed according to the method described by Jian [13] and Chang [14]. In brief, the collected worms were cleared in lactophenol. Then the parasites were fixed and examined with an optical microscope and by scanning electron microscopy (SEM) to confirm the characteristic structures, including the buccal cavity, and bursa and dorsal rays on the copulatory bursa in male worms.

The mitochondrial *cox*1 gene was used to molecularly identify the hookworms [15]. Total genomic DNA was extracted from individual specimens using the EasyPure Genomic DNA Kit (TransGen Biotech, China). The *cox*1 gene was amplified by polymerase chain reaction (PCR) using the primer combination reported by Zhan [16]: forward primer (NaF, 5′-TTCGTTTGGAGTTGGCT) and reverse primer (NaR, 5'-TAGCTCCAGCCAAAACT). The amplification profile consisted of initial denaturation for 1 min at 95 °C, followed by 35 cycles of 60 s at 95 °C, 60 s at 55 °C, and 120 s at 72 °C. The PCR products were purified using the EasyPure PCR Purification Kit (TransGen Biotech, China) and sequenced in both directions by GENEWIZ (Suzhou, China). Sequence similarity analysis was conducted through a BLAST search (http://blast.ncbi.nlm.nih.gov/Blast.cgi).

### Literature review of cases from Henan Province

Although previous literature reviews of Chinese cases of ancylostomiasis have been performed, no reports have focused on ancylostomiasis in Henan Province. In addition, because most clinical reports have been published in Chinese, the epidemiological data have not been accessible to a large part of the international scientific community. Therefore, on the basis of previous work, we updated knowledge about the prevalence of ancylostomiasis in Henan Province. In this study, we searched the PubMed, China National Knowledge Infrastructure (CNKI), Chinese Science and Technology Periodical (VIP), and Wanfang databases to collect relevant data. We reviewed studies cited in articles that were identified from October 1, 1949, to December 31, 2020. The cases of hookworm infection are divided into two categories, namely epidemiological cases and hospital-diagnosed cases, according to the standard described by Li [5].

### Mitogenome sequencing and phylogenetic analysis

The whole mt genome of one *N. americanus* isolate collected from the patient was amplified in two overlapping fragments by long PCR using primers and methods described by Hu [10]. The two overlapping products were mixed in approximately equal amounts after determining the concentration of each amplicon. The library was prepared using the TruSeq DNA PCR-Free Sample Preparation Kit (Illumina, USA) following the manufacturer’s protocols. The DNA concentrations of all purified libraries were quantified, and 30 ng of each were pooled together. The oligonucleotide mix was sequenced on an Illumina HiSeq 2000 at GENEWIZ (Beijing, China). The quality of the original reads was evaluated using the FastQC v.0.11.5 tool, and all ambiguous nucleotides and reads with an average quality value (lower than Q20) were excluded. The trimmed sequences were mapped against a reference mt genome of *N. americanus* (NC_003416) using the CLC Genomic Workbench v.7.0.4 (Qiagen, Germany). Contigs with hits to mitochondrial genes or genomes were identified and extracted from the CLC Genomic Workbench. A contig identified as mt genome was manually examined for repeats at the beginning and end of the sequence to establish a circular mtDNA. It was then annotated using MITOS followed by manual validation of the coding regions using the NCBI ORF Finder (http://www.ncbi.nlm.nih.gov/gorf/gorf.html) [17].

To reconstruct the phylogeny of hookworms, all available mt genomes of hookworms in the GenBank database were collected. To date (June 30, 2021), 14 fully sequenced mt genomes have been published, representing three genera: *Ancylostoma* (eight mtDNAs), *Bunostomum* (four), and *Necator* (two). *Strongylus equinus* (KM605251) and *S. vulgaris* (AP017698) were used as an outgroup. The 12 protein-coding genes (PCGs) of mt were used to construct the phylogenetic trees using maximum likelihood (ML) and Bayesian inference (BI) methods. PCGs were separately aligned using MEGA v.7 [18], then combined using MAFFT v7 [19]. The best-fit nucleotide substitution model was selected by jModelTest 2 under the Akaike information criterion (AIC) [20]. The ML analysis was performed in MEGA v.7 with 1000 bootstrap replications. BI was performed in MrBayes v.3.2 [21]. The analysis consisted of two runs, each with four Markov chain Monte Carlo (MCMC) chains running for 5,000,000 generations, and sampling every 100th generation.

## Results and discussion

**Fig. 1:**
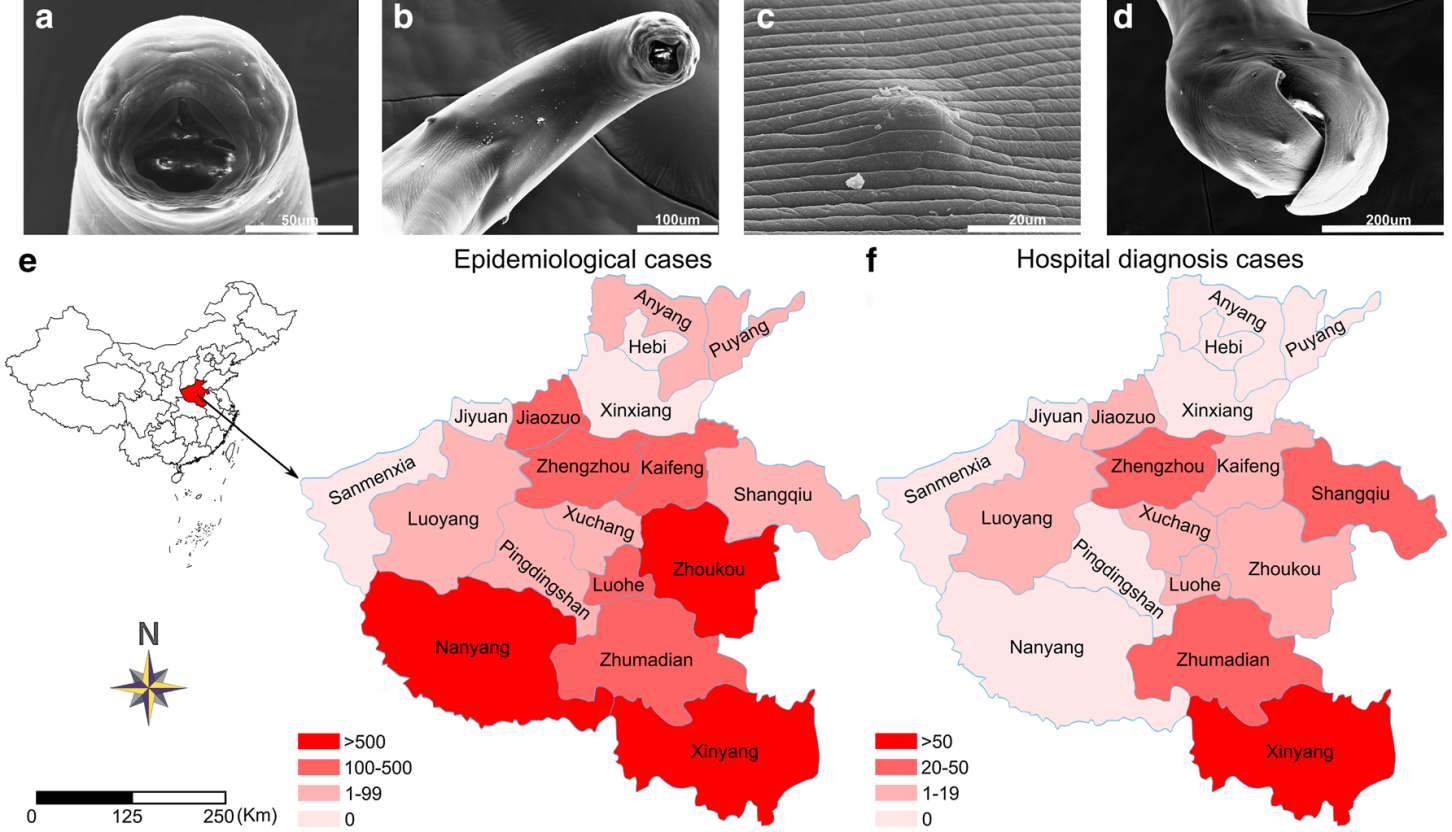
Scanning electron microphotographs of *Necator americanus* clinical isolate collected in this study and geographical distribution of epidemiological and hospital cases of ancylostomiasis in Henan Province in central China. **a** Buccal cavity with cutting plate. **b** A pair of neck papillae on the ventrolateral margin. **c** Transverse cuticular striations on the body surface. **d** Copulatory bursa with several papillae on the ventral base. **e** Epidemiological cases of ancylostomiasis in Henan Province. **f** Hospital-diagnosed cases of ancylostomiasis in Henan Province

**Fig. 2:**
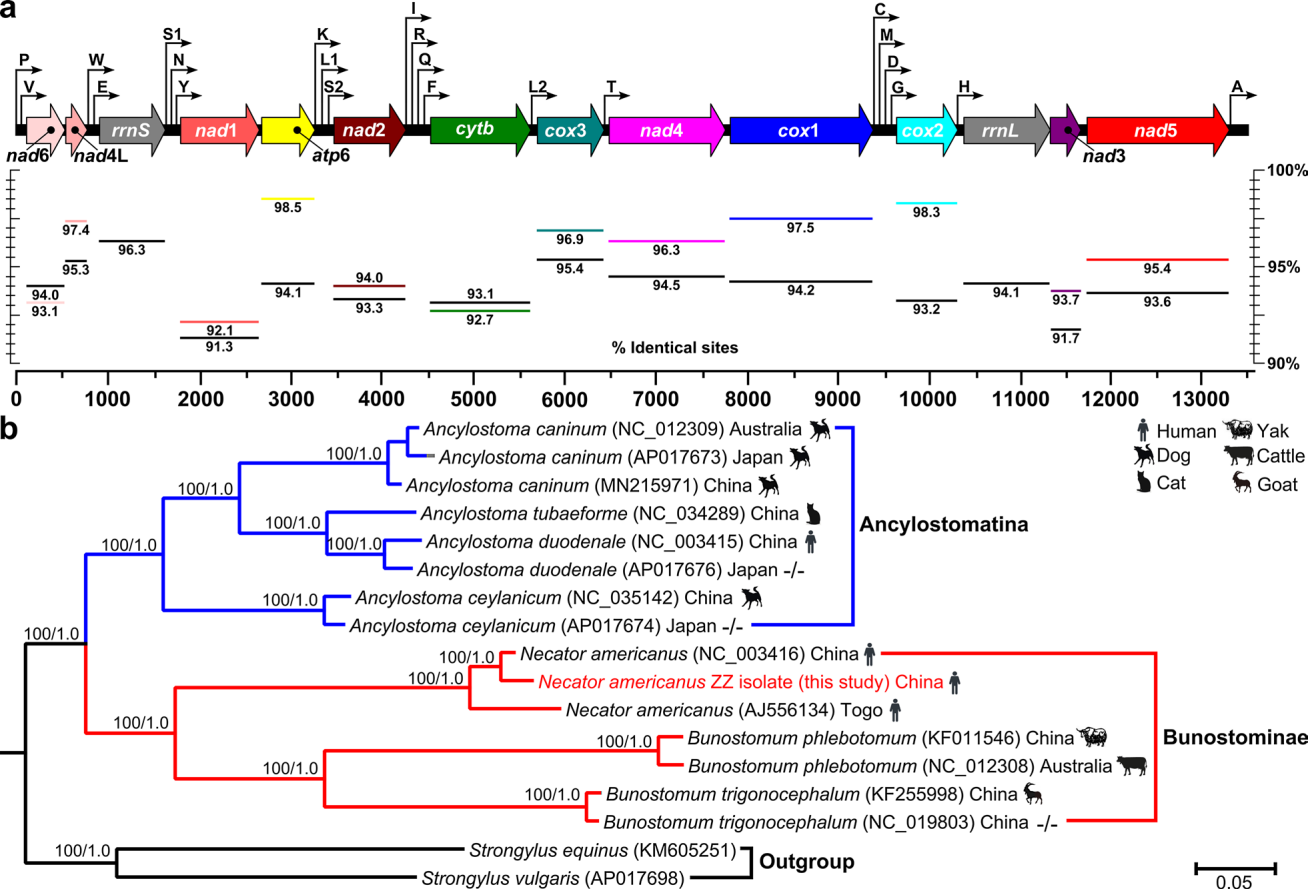
**a** Linear maps of the mitochondrial genomes of *Necator americanus*. Outline arrows indicate the positions and direction of transcription of protein- and rRNA-coding genes; hairline arrows indicate the position of tRNA-coding genes (trn). Black solid horizontal lines and colored horizontal lines below depict average nucleotide and amino acid identities of aligned protein and rRNA coding among three *N. americanus* isolates from China (NC_003416 and this study) and Togo (AJ556134). *atp*6, adenosine triphosphatase subunit 6; *cox*, cytochrome c oxidase complex; *cyt*b, cytochrome b; *nad*, nicotinamide dehydrogenase complex; *rrn*L, large subunit rDNA; *rrn*S, small subunit rDNA. **b** The phylogenetic tree of 14 available mt genomes of hookworms in the GenBank database to date (June 30, 2021) using ML and BI methods based on 12 protein-coding genes. Numbers above branches represent the bootstrap values and Bayesian posterior probabilities. Only bootstrap values above 60 and posterior probabilities above 0.6 are shown

The patient, female, 68 years old, lived in the suburbs of Zhengzhou city, Henan Province, in central China (34°38′ N, 113°11′ E). In January 2019, the woman was admitted to the First Affiliated Hospital of Zhengzhou University complaining of a bitter taste in her mouth and upper abdominal discomfort for more than 6 months. The patient had a history of hypertension and coronary sclerosis. Physical examination showed no obvious symptoms or signs, and her vital signs were stable. Initial laboratory studies showed that her levels of red blood cells, hemoglobin, hematocrit, mean corpuscular hemoglobin, and iron were low, whereas platelets and the blood sedimentation rate were elevated, suggesting iron deficiency anemia. The patient complained that she often worked barefoot in the fields. Gastroduodenoscopy analysis showed several active nematodes in the duodenal bulb (Additional file 1: Figure S1). The worms were collected by endoscopic forceps and identified as *N. americanus* according to morphological characteristics. More specifically, (1) *N. americanus* has a slightly rounded buccal cavity provided with a pair of ventral semilunar cutting plates and a pair of smaller dorsal cutting plates (Fig. 1a); (2) a pair of conspicuous neck papillae were present on the ventrolateral margin (Fig. 1b); (3) there were transverse cuticular striations on the body surface (Fig. 1c); (4) the posterior end of the hookworm was rounded and exhibited a copulatory bursa with several papillae on its ventral base (Fig. 1d); and (5) the distal end of the copulatory bursa is divided into two branches, and each branch is further divided into three small rays (Additional file 2: Figure S2). In addition, the *cox*1 sequences of collected hookworms in this study were identified. All worms generated the identical sequence. The comparison results indicated that the *cox*1 sequence of hookworms here had a 99.83% similarity with that of the published *N. americanus* from Yunnan Province in China. For the treatment of ancylostomiasis, the patient was given oral albendazole tablets (GSK Pharmaceuticals Inc., Tianjin, China) 200 mg three times a day for 1 month. During this period, iron and recombinant human erythropoietin were continuously supplemented. The stool occult blood test after treatment showed negative results, and no hookworm eggs were found in the Kato-Katz test. The concentration of serum hemoglobin reached a normal value 2 months after the therapy.

Henan Province has an area of approximately 167,000 km^2^, with a population of approximately 100 million people. A total of 25,948 cases of hookworm infection in Henan Province were reported from 1949 to 2020, including 25,631 cases (98.78%) from epidemiological investigations and 317 patients (1.22%) diagnosed at hospitals (Fig. 1e, f and Additional file 3: Table S1). Among all reported ancylostomiasis, 2596 cases were caused by *N. americanus*, and 11,869 cases were caused by *A. duodenale*. Only one case was reported by simultaneous infection. Most of the reported cases were distributed in southern areas of Henan Province, such as Nanyang and Xinyang. However, no cases have ever been reported in four cities: Hebi, Xinxiang, Sanmenxia, and Jiyuan. According to an analysis by 5-year phases, hospital-diagnosed cases displayed an increasing trend each year from 1949 to 2015 (Additional file 4: Figure S3), but have declined dramatically in recent years (2016–2020), with only four cases reported. For epidemiological cases, from 1949 to 1970, few hookworm infection cases were reported (271 cases). During 1986–1990, the reported ancylostomiasis cases increased dramatically and reached 19,466 cases. However, the number of cases began to decrease year by year after 1990; in particular, in the past 5 years, only 96 epidemiological cases have been reported. Since the first national survey on the distribution of human parasites was carried out in 1990 in China, many provinces including Henan have carried out comprehensive measures for the prevention and control of parasitic diseases in rural areas, focusing on deworming treatment, combined with health education and fecal management [22, 23]. Therefore, the number of cases of ancylostomiasis began to decline significantly after 1990. Generally, although ancylostomiasis cases have dropped substantially in recent years, hookworm infection is still a public health problem that negatively influences health and hinders socioeconomic development in underdeveloped areas and remote rural areas of Henan Province. Positive measures for preventing and controlling ancylostomiasis such as improving the level of knowledge about people’s health and alleviating health poverty are required by both health services and individuals.

In recent years, the genotyping of hookworms based on suitable molecular markers has played an important role in the modern diagnosis and epidemiological investigation of hookworms, and the mt genome can provide more potential markers for molecular identification. In this study, a total of 1,024,362 sequence reads were mapped to the reference mitochondrial genome of *N. americanus* [10]. The mt genome of the *N. americanus* collected here was fully annotated and encoded 12 PCGs (*cox*1-3, *nad*1-6, *nad*4L, *atp*6, and *cyt*b), two ribosomal RNA (*rrn*) genes, and 22 transfer RNA genes, but lacked an *atp*8 gene (Fig. 2a). All 36 genes were encoded in identical order and direction, in accordance with other sequenced mt genomes of hookworms [11, 12, 24]. Among the 12 PCGs, the most common initiation codon was ATT (for eight genes), followed by TTG (three genes). Eight PCGs were predicted to have a TAA or TAG termination codon. The remaining protein genes were inferred to end with an abbreviated stop codon, such as T or TA. Incomplete termination codons are common in the mitochondrial genomes of parasitic helminths [24]. Both *rrn*S and *rrn*L were 699 bp and 957 bp in length, and located in the positions between tRNA-Glu and tRNA-Ser and between tRNA-His and *nad*3, respectively. The lengths of the 22 tRNA genes ranged from 52 (tRNA-Ser) to 62 (tRNA-Lys) nucleotides (nt). Among the *N. americanus* isolates (NC_003416, AJ556134 and this study), the full-length mt genomes were identical in 93.4% nucleotides with the alignment of 13,623 bp in length. Comparing the PCGs, *N. americanus* isolates displayed high levels of sequence conservation ranging from 91.3% of identical nucleotides in *nad*1 to 95.4% in *cox*3. For *cox*1, the nucleotide identity reached 97.5%. The amino acid sequence similarities ranged from 92.1% (*nad*1) to 98.5% (*atp*6). The results of the sequence divergence of PCGs suggest that *nad*1 shows high sequence variability among isolates, indicating that this gene is suitable as a genetic marker for population genetics studies of *N. americanus* of different geographical origins. The mt large and small subunit rDNAs (*rrn*L, *rrn*S) shared 94.1% and 96.3% identical nucleotides, respectively.

The likelihood models identified by the jModelTest (AIC) suggested that the GTR + G + I model was most suitable for included data partitions. Basal identical tree topologies were generated through phylogenetic inference based on both ML and BI methods. The phylogenetic pattern is demonstrated in Fig. 2b. Hookworm isolates collected here were grouped into two obvious clades: one clade included species of *A. caninum*, *A. tubaeforme*, *A. duodenale*, and *A. ceylanicum* corresponding to the subfamily of Ancylostomatinae; the other clade contained species of *N. americanus*, *Bunostomum phlebotomum*, and *B. trigonocephalum* corresponding to the subfamily of Bunostominae. Within the Bunostominae, three *N. americanus* isolates formed a single group and as a sister group with the *Bunostomum* species, consistent with a recent molecular study [24]. Among *N. americanus* isolates, isolates from China were more closely related to each other than to that from Togo, indicating that long-distance geographical isolation might accelerate the genetic differences among *N. americanus* isolates.

## Conclusions

Scanning electron microscopy is useful for examining key characteristics of hookworms. The mt genome generated by NGS technology is accurate and reliable, and represents a powerful tool for the identification of hookworms. The *nad*1 gene showed high sequence variability among isolates, which is worth considering for future population genetics studies. The mitogenomics comparative analysis supports the monophyly of hookworm isolates from different hosts and distinct geographical locations. *Necator americanus* isolates from China were more closely related to each other than to that from Togo, indicating that long-distance geographical isolation might accelerate the genetic differences among *N. americanus* isolates. According to a review of ancylostomiasis in central China over the past 72 years (1949–2020), ancylostomiasis cases have declined substantially in recent years; however, hookworm disease is still a public health problem that negatively influences health and hinders socioeconomic development in underdeveloped areas and remote rural areas.

## Supplementary Information

**Additional file 1: Figure S1.** Hookworm infections in the patient’s small bowel, as observed with endoscopy.

**Additional file 2: Figure S2.** Morphological characteristic of dorsal ray of *Necator americanus.*

**Additional file 3: Table S1.** Geographical distribution of ancylostomiasis in Henan Province of central China.

**Additional file 4: Figure S3.** Epidemiological investigation and hospital-diagnosed hookworm cases for 5-year periods from 1949 to 2020 in Henan Province of central China.

## Data Availability

The data supporting the results of this paper are included in the paper.

## Abbreviations

AIC: Akaike information criterion
BI: Bayesian inference
ML: Maximum likelihood
NGS: Next-generation sequencing
PCGs: Protein-coding genes
