# Supplementary material for: Hookworm infection in central China: morphological and molecular diagnosis
Source: Parasit Vectors. 2021 Oct 14;14:537. doi: 10.1186/s13071-021-05035-3 (PMC8518228; doi:10.1186/s13071-021-05035-3)
Supplement: Supplementary file 1 — Additional file 1: Figure S1. Hookworm infections in the patient’s small bowel, as observed with endoscopy. [file 13071_2021_5035_MOESM1_ESM.doc]

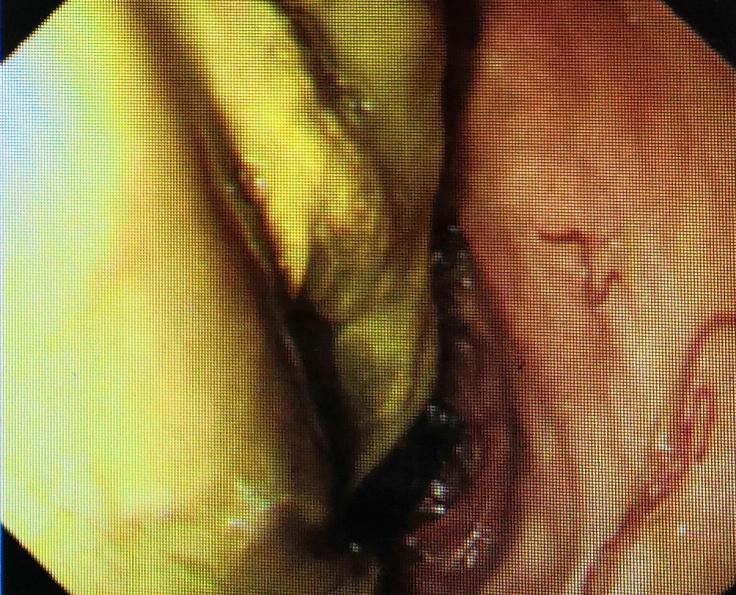


**Figure S1.** Hookworm infections in the patient’s small bowel, as observed with endoscopy. Hookworms appeared with reddish bodies attached to the intestinal mucosa.
