# Supplementary figures and images for: Hookworm infection in central China: morphological and molecular diagnosis
Source: Parasit Vectors. 2021 Oct 14;14:537. doi: 10.1186/s13071-021-05035-3 (PMC8518228; doi:10.1186/s13071-021-05035-3)

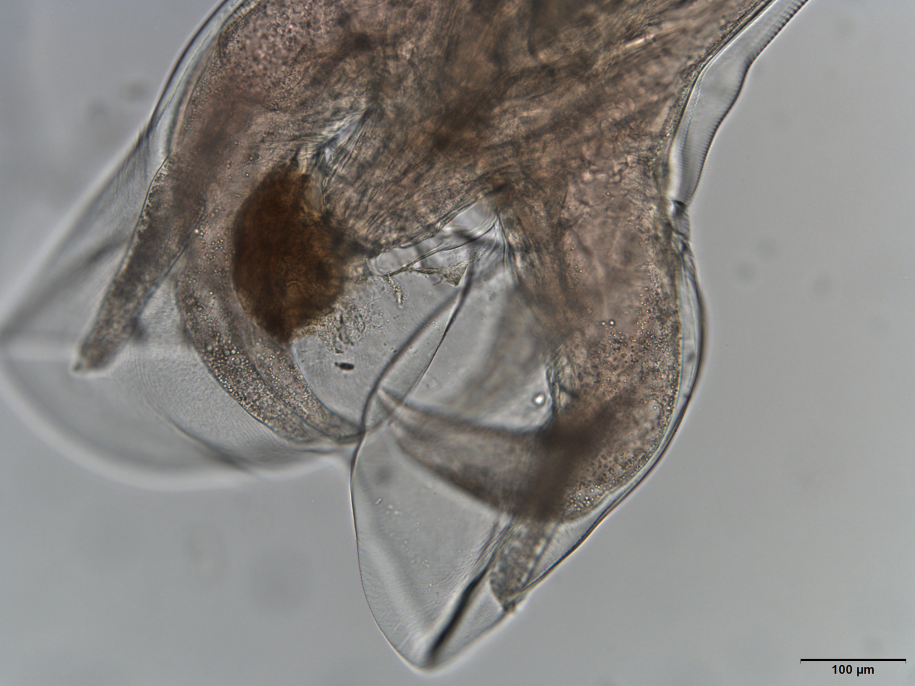


**Figure S2.** Morphological characteristic of dorsay ray of *Necator americanus.*

Supplement: Supplementary file 2 — Additional file 2: Figure S2. Morphological characteristic of dorsal ray of Necator americanus. [file 13071_2021_5035_MOESM2_ESM.doc]
