# Supplementary material for: Hookworm infection in central China: morphological and molecular diagnosis
Source: Parasit Vectors. 2021 Oct 14;14:537. doi: 10.1186/s13071-021-05035-3 (PMC8518228; doi:10.1186/s13071-021-05035-3)
Supplement: Supplementary file 3 — Additional file 3: Table S1. Geographical distribution of ancylostomiasis in Henan Province of central China. [file 13071_2021_5035_MOESM3_ESM.doc]

**Table S1.** Geographical distribution of ancylostomiasis in Henan province of central China.

^1^It represents hookworm disease caused by *Necator americanus*;

| Years | Number of cases | | | | Total | References |
| --- | --- | --- | --- | --- | --- | --- |
|  | Na^1^ | Ad^2^ | Mix^3^ | Unk^4^ |  |  |
| 1949-1955 | 0 | 0 | 0 | 270 | 270 | [1-71] |
| 1956-1960 | 0 | 0 | 0 | 0 | 0 |  |
| 1961-1965 | 0 | 0 | 0 | 1 | 1 |  |
| 1966-1970 | 0 | 0 | 0 | 0 | 0 |  |
| 1971-1975 | 0 | 0 | 0 | 16 | 16 |  |
| 1976-1980 | 0 | 0 | 0 | 2579 | 2579 |  |
| 1981-1985 | 0 | 1 | 0 | 0 | 1 |  |
| 1986-1990 | 2512 | 11473 | 1 | 5480 | 19466 |  |
| 1991-1995 | 0 | 0 | 0 | 100 | 100 |  |
| 1996-2000 | 0 | 1 | 0 | 680 | 681 |  |
| 2001-2005 | 11 | 202 | 0 | 1963 | 2176 |  |
| 2006-2010 | 0 | 73 | 0 | 104 | 177 |  |
| 2011-2015 | 0 | 119 | 0 | 262 | 381 |  |
| 2016-2020 | 73 | 0 | 0 | 27 | 100 |  |
| Total | 2596 | 11869 | 1 | 11469 | 25948 |  |

^2^It represents hookworm disease caused by *Ancylostoma duodenale*;

^3^It represents hookworm disease caused by *Necator americanus* and *Ancylostoma duodenale*;

^4^Itrepresents the unknown type of hookworm that causes hookworm disease.

**References**

[1] Wang Y, Wang Y, Wang Y (2020) The role of soil nematodes in the development of soil nematodes in China. Bulletin of Disease Control & Prevention(China), 35(06):23-25. (in Chinese)

[2] Du L, Zhang JJ (2019) Epidemiology of important human intestinal worms in PingDingshan City, Henan Province. Journal of Tropical Diseases and Parasitology, 17(04):218-221. (in Chinese)

[3] Chen WQ, Li SH, Zhang YL et al (2019) Epidemiological status and influencing factors of intestinal parasitic diseases in rural children in Henan Province. Chinese Journal of Schistosomiasis Control, 31(05):491-497. (in Chinese)

[4] Zhu HH, Zhou CH, Zu TJ et al (2020) Survey of soil-derived nematode infection in urban residents in China in 2015.Chinese Journal of Schistosomiasis Control, 32(05):476-482. (in Chinese)

[5] Zhu HH, Huang JL, Zhu TJ et al (2019) Surveillance data of soil-derived nematode infection in China in 2017.Chinese Journal of Parasitology and Parasitic Diseases, 37(01):12-17. (in Chinese)

[6] Wang KM, Pan SG, Zhang X et al (2018) Epidemiological analysis of hookworm infection among middle-aged and elderly people in Yuanhui district of Luohe city. Henan Journal of Preventive Medicine, 29(05):357-358 . (in Chinese)

[7] Xu BL, Zhang HW, Deng Y et al (2018) Analysis on the results of stratified sampling and influencing factors of human parasitic diseases in Henan Province. Chinese Journal of Epidemiology, 39(03):322-328. (in Chinese)

[8] Yang CY, Lu DL, Zhang YL et al (2018) The status of human intestinal parasites in the ecological zone of Yanshan-Taixing Mountain in 2015. China Tropical Medicine, 18(01):84-88. (in Chinese)

[9] Zhang YL, Zhu YK, Chen WQ et al (2018) Investigation on human intestinal worm infection in urban areas of Henan Province in 2015.Chinese Journal of Parasitology and Parasitic Diseases, 36(02):135-138. (in Chinese)

[10] Deng Y, Zhang YL, Chen WQ et al (2017) Investigation on the status of human intestinal parasitic infection in eastern Henan province in 2015.Chinese Journal of Parasitology and Parasitology, 35(05):450-455. (in Chinese)

[11] Li QP, Li FK, Jia WL et al (2017) Investigation on human soil-derived nematode infection in Wancheng district of Nanyang city in 2015.Henan Journal of Preventive Medicine, 28(08):605-606. (in Chinese)

[12] Chen WQ, Zhang YL, Li SH et al (2017) Monitoring results of national soil-derived nematode disease surveillance site in Huaiyang County, Henan Province from 2006 to 2015.Chinese Journal of Schistosomiasis Control, 29(05):583-586. (in Chinese)

[13] Zhang YL, Zhu YK, Chen WQ et al (2017) Investigation and analysis of human major parasitic diseases in Huaiyang hilly ecological area, Henan Province in 2015.Chinese Journal of Schistosomiasis Control, 29(05):607-611. (in Chinese)

[14] Zhang ML, Wang L, Li JL (2017) Anchylostomia infection in a family of four people in three generations. Practical Journal of Medicine & Pharmacy, 34(06):529-531. (in Chinese)

[15] Li TP, Li TP, Du YF et al (2017) Survey and analysis of soil-derived nematodes in Zhumadian City, China in 2015.Henan Journal of Preventive Medicine, 28(02):128-131. (in Chinese)

[16] Deng TF (2014) Investigation on soil-derived nematode infection in humans in Shangqiu city, 2010-2012. Chinese Journal of Vector Biology and Control, 25(05):478-479. (in Chinese)

[17] Ma X (2013) Surveillance of soil-derived nematodes in Luohe City, 2012. Henan Journal of Preventive Medicine, 24(04):292-293. (in Chinese)

[18] Liu PX, Liu Y, Liu X (2013) Monitoring results of soil-derived nematodes in children under 12 years of age in Jiaozhuo, Henan Province, 2012.Disease Surveillance, 28(03):245-246. (in Chinese)

[19] Liu L, Wen J (2012) nvestigation on intestinal parasitic infection of children in a hospital in Zhumadian in 2011. International Journal of Laboratory Medicine, 2012,33(20):2451-2452 . (in Chinese)

[20] Chen WQ, Lin XM, Yan QY et al (2010) Analysis of surveillance results of soil-derived nematode disease in Henan Province in 2009.Journal of Pathogen Biology, 5(11):863-864. (in Chinese)

[21] Li Y, Li TP (2010) Investigation on the status of soil-derived nematode infection in Zhumadian City, China in 2009.Henan Journal of Preventive Medicine 21(05):381-382. (in Chinese)

[22] Yuan Z, Dai YW (2010) Endoscopic diagnosis of duodenal ancylostomiasis in 72 cases. Chinese Community Doctors, 12(27):188. (in Chinese)

[23] Xu CH, Zhao H, Zhao HX et al (2010) The effect of soil nematode infection on the quality of soil nematode in China. Bulletin of Disease Control & Prevention(China), 25(03):32. (in Chinese)

[24] Sun YR, Xi JW, Yao HX et al (2010) Intestinal parasitic infection in patients in a hospital in Zhengzhou. Journal of Tropical Medicine, 10(05):563-566. (in Chinese)

[25] Liu CJ, Yao DS, Lin XM et al (2009) Monitoring results of soil-derived nematode infection in Henan Province in 2008. Journal of Pathogen Biology, 4(10):774-776. (in Chinese)

[26] Wang RJ, Qi M, Zhao YF et al (2009) Intestinal parasitic infection in children in Linzhou, Henan Province. Journal of Tropical Medicine, 9(10):1184-1187. (in Chinese)

[27] Li YX, Xie WB, Hao JL et al (2009) Monitoring results of soil-derived nematode disease in Zhou Kou city in 2006.Journal of Preventive Medicine Intelligence, 25(06):473-474. (in Chinese)

[28] Du MJ, You CX, Zhu X et al (2009) Analysis on the prevalence of intestinal parasitic diseases in Luoyang city. Chinese Journal of Disease Control & Prevention, 13(03):375-376. (in Chinese)

[29] Wang Q, Zhou Y, Lu B et al (2009) Intestinal Parasite Infection in Clinical Patients in Kaifeng, Henan Province. Journal of Tropical Medicine, 9(05):510-514. (in Chinese)

[30] Yu YY, Hu XJ, Gao P et al (2007) Intestinal Parasite Infection in Anyang City, Henan Province. Journal of Pathogen Biology, (02):92. (in Chinese)

[31] Xu BL, Zhang HW, Zhao XD et al (2006) Investigation of intestinal parasitic infection and its socio-economic factors in Henan Province. International Journal of Medical Parasitic Diseases, (04):174-176. (in Chinese)

[32] Xu BL, Zhao XD, Su YP et al (2005) Investigation and analysis of the epidemic status of major human parasitic diseases in Henan Province. Journal of Pathogen Biology, (06):454-457. (in Chinese)

[33] Shen DY, Yang J, Yang GY et al (2005) Current situation and analysis of intestinal nematode infection in Xinyang city. Journal of Pathogen Biology, (05):87. (in Chinese)

[34] Li H, Xu BL, Zhao XD et al (2005) Epidemiology of important parasitic diseases in the population of Zhou Kou in 2002. Preventive Medicine Tribune, (05):528-530. (in Chinese)

[35] Wang YH, Huang HM (2005) Intestinal parasitic infection among students in a school in Henan province for 12 years. Chinese Journal of Parasitology and Parasitic Diseases, (03):192. (in Chinese)

[36] Cui JH (2005) Analysis of six cases of intestinal ancylostomiasis misdiagnosed. Clinical Misdiagnosis & Mistherapy, (05):305. (in Chinese)

[37] Wang WS, Chen GY, Zhang HP et al (2005) Investigation of two cases of human intestinal parasitic diseases in Neixiang county, Henan province. Journal of Pathogen Biology, (02):126. (in Chinese)

[38] Chen KH, Kang JH, Mei HX et al (2004) 30 cases of hookworm disease were confirmed by gastroscopy . Clinical Misdiagnosis & Mistherapy, (10):740. (in Chinese)

[39] Fan EJ, Yang Y, Jia SW et al (2004) Investigation on important human parasitic infection in Weishi county. Henan Journal of Preventive Medicine, (05):304. (in Chinese)

[40] Li H, Lin XM, Huang Q et al (2004) Analysis of intestinal parasitic infection in Henan province. Journal of Pathogen Biology, (04):37-39. (in Chinese)

[41] Fei L (2004) The role of the Chinese government in the prevention of parasitic diseases in China. Chinese Journal of School Doctor, (04):352. (in Chinese)

[42] Xu XL, Wu Y, Lu DL et al (2003) Investigation on human intestinal parasitic infection in the suburb of Jiaozuo. Journal of Pathogen Biology, (05):80. (in Chinese)

[43] Li Q, Li MX, Wei HM (2003) Investigation on the status of human intestinal parasitic diseases in rural areas of Neixiang County, Henan Province. Parasitoses and Infectious Diseases, (03):114. (in Chinese)

[44] Wang YH (2002) Investigation and comparison of two cases of intestinal parasites infection. Journal of Zhengzhou Railway Vocational and Technical College, (03):63-64. (in Chinese)

[45] Song HR (2002) Analysis of 12 cases of misdiagnosis of hookworm anemia. Chinese Journal of Practical Medicine, (05):63. (in Chinese)

[46] Niu AO, Fang ZM (1999) Gastroscopic pathology confirmed a case of hookworm disease. Chinese Journal of Parasitology and Parasitic Diseases, (01):37. (in Chinese)

[47] He LJ, Yan QY, Shi HF et al (1998) Investigation on the status of intestinal parasitic infection in suburban population of Kaifeng. Henan Medical Research, (01):56-58. (in Chinese)

[48] Wang RG, Yan KC, Sun JZ et al (1996) Intestinal parasitic infection in rural areas of Neixiang county, Henan province. Parasitoses and Infectious Diseases, (04):159. (in Chinese)

[49] Chang J, Shang LY, Zhao QF et al (1994)Investigation and analysis on the distribution of human parasites in Henan Province. Henan Journal of Preventive Medicine, (05):249-252. (in Chinese)

[50] Cui ZL, Zhang YL, Sun JZ(1994) The distribution of soil-derived nematodes in the northeast plain of Henan Province. Chinese Journal of Parasitology and Parasitic Diseases, (S1):184-186. (in Chinese)

[51] Liu H, He LJ, Yan QY(1994) Investigation on parasitic infection in population in northwest mountainous area of Henan Province. Chinese Journal of Parasitology and Parasitic Diseases, (S1):193-194. (in Chinese)

[52] Liu BL, Zhang LM, Cheng JL(1992) A case of hookworm gastrointestinal hemorrhage misdiagnosed. Clinical Misdiagnosis & Mistherapy, (04):184. (in Chinese)

[53] Liu YH, Zhu DS(1990) A case of acute gastrointestinal massive hemorrhage caused by intestinal hookworm disease. Chinese Journal of Applied Clinical Pediatrics, (01):10. (in Chinese)

[54] Lu JZ (1989) Investigation of intestinal worm infection in rural residents in Jiaozuo, Henan Province. Journal of Pathogen Biology, (02):118-119. (in Chinese)

[55] Zhang KR, Qu MQ, Yin QY et al (1989) Investigation of intestinal parasitic infection in Kaifeng city. Journal of Zhengzhou University(Medical Sciences), (02):128-130. (in Chinese)

[56] Zhang Y, Wang Y, Wang Y (2012) The role of the pathogen in the pathogenesis of intestinal parasites in China. Chinese Journal of Parasitology and Parasitic Diseases, 36 (2): 356. (in Chinese)

[57] Gao YZ(1965) A case report of hookworm disease in infant.Journal of Zhengzhou University (Medical Sciences), (01):51. (in Chinese)

[58] Wang YZ, Yin QY, Jin XX et al (1984) Intestinal parasitic parasites in the population of Henan Province. Journal of Zhengzhou University(Medical Sciences), (02):22-24. (in Chinese)

[59] Peng JH (2015) Investigation on soil-derived nematode infection in XuChang, Henan Province in 2012. Chinese Journal of Parasitology and Parasitic Diseases, 33(05):395-396. (in Chinese)

[60] Zhao YC, Zhao J, Cheng JM (2012) Investigation on soil-derived nematode disease in Gutong village of Luohe city in 2011. China Rural Health, (Z2):478. (in Chinese)

[61] Liu SH, Zhang L, Huang HQ et al (2012) Surveillance of soil-derived nematode disease in Xinyang City, Henan Province, 2010. Occupation and Health, 28(17):2130-2132. (in Chinese)

[62] Fan YL, Hu WH (2011) Analysis of nine cases of upper gastrointestinal bleeding caused by hookworm disease. Clinical Misdiagnosis & Mistherapy, 24(Z1):22-23. (in Chinese)

[63] Kang JH (2015) Electronic gastroscope diagnosis of duodenal clinical analysis of 30 cases of hookworm disease. Chinese Journal of Trauma and Disability Medicine, (2) : 167-168 . (in Chinese)

[64] Yuan Z (2013) 79 cases of duodenal hookworm disease were diagnosed by gastroscopy. Chinese Journal of Practical Medicine, 35 (1): 87-91. (in Chinese)

[65] Zhang ML, Xing XP (2008) A case of duodenal hookworm confirmed by gastroscopy and microscope. Chinese Journal of Laboratory Medicine, 31(7):821-822.. (in Chinese)

[66] Ye SM (1987) A case of neonatal hookworm disease. Chinese Journal of Pediatrics, 25(2):107. (in Chinese)

[67] Lu D, Li P, Song JD (1994) Intestinal parasites of population in mountainous and hilly areas of southern Henan province, Henan province. Chinese Journal of Parasitology and Parasitic Diseases, (S1):190-192. (in Chinese)

[68] Li DL, Lu DL (2011) Infectious Parasite Infection in Children in Henan Province. Henan Journal of Preventive Medicine, 22(06):431-434. (in Chinese)

[69] Liu W, Qu WH (1995) Clinical analysis of 24 cases of anemia caused by hookworm disease in children. Journal of Medical Forum, 199527. (in Chinese)

[70] Zhang XD, Hu HQ, Tao YW (1995) Analysis of 21 cases of misdiagnosis of hookworm disease. Practical Journal of Medicine & Pharmacy, 1995310. (in Chinese)

[71] Lin XM, Chang J, Li GL (1994) Investigation on the distribution of human parasites in Nanyang Basin, Henan Province. Chinese Journal of Parasitology and Parasitic Diseases, (S1):187-189. (in Chinese)
