# Supplementary material for: Hookworm infection in central China: morphological and molecular diagnosis
Source: Parasit Vectors. 2021 Oct 14;14:537. doi: 10.1186/s13071-021-05035-3 (PMC8518228; doi:10.1186/s13071-021-05035-3)
Supplement: Supplementary file 4 — Additional file 4: Figure S3. Epidemiological investigation and hospital-diagnosed hookworm cases for 5-year periods from 1949 to 2020 in Henan Province of central China. [file 13071_2021_5035_MOESM4_ESM.doc]

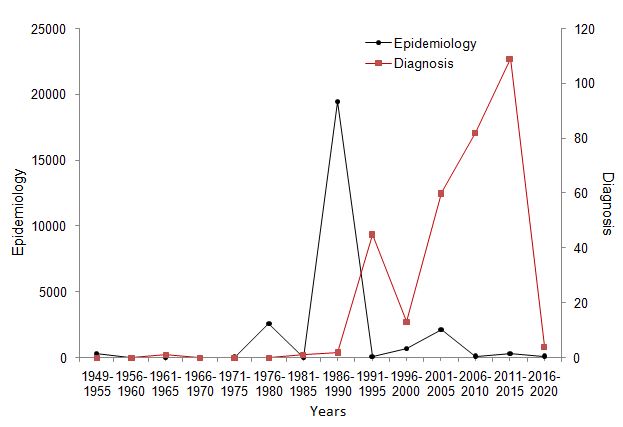


**Figure S3.** Epidemiological investigation and hospital-diagnosed hookworm cases for 5-year periods from 1949 to 2020 in Henan Province of central China.
